# Characterization of Immune-Related Long Non-coding RNAs to Construct a Novel Signature and Predict the Prognosis and Immune Landscape of Soft Tissue Sarcoma

**DOI:** 10.3389/fcell.2021.709241

**Published:** 2021-09-24

**Authors:** Zhengjun Lin, Ke Pang, Hongli Li, Xianghong Zhang, Jia Wan, Tao Zheng, Tang Liu, Weijun Peng

**Affiliations:** ^1^Department of Orthopedics, The Second Xiangya Hospital, Central South University, Changsha, China; ^2^Xiangya School of Medicine, Central South University, Changsha, China; ^3^Department of Integrated Traditional Chinese and Western Medicine, The Second Xiangya Hospital, Central South University, Changsha, China

**Keywords:** soft tissue sarcomas, immune-related lncRNAs, prognostic signature, immune microenvironment, chemosensitivity

## Abstract

**Background:** Increasing evidence has demonstrated that immune-related long non-coding RNAs (irlncRNAs) are critically involved in tumor initiation and progression and associated with the prognosis of various cancers. However, their role in soft tissue sarcoma (STS) remains significantly uninvestigated.

**Materials and Methods:** Gene expression profiles were extracted from The Cancer Genome Atlas (TCGA) and Genotype-Tissue Expression (GTEx) for the identification of irlncRNAs. Univariate analysis and modified least absolute shrinkage and selection operator (LASSO) penalized regression analysis were employed to determine differently expressed irlncRNA (DEirlncRNA) pairs of prognostic value, and subsequently, a risk signature based on DEirlncRNA pairs was established. Furthermore, Kaplan–Meier analysis and the area under the receiver operating characteristic curve (AUC) were used to assess survival differences and the predictive accuracy of the risk signature, respectively. Lastly, the correlation of irlncRNAs with immune characteristics and chemosensitivity in patients with STS were further investigated.

**Results:** A total of 1088 irlncRNAs were identified, and 311 irlncRNAs were distinguished as DEirlncRNAs. A total of 130 DEirlncRNA pairs were further identified as prognostic markers, and 14 pairs were selected for establishing a risk signature. The irlncRNA-based risk signature functioned as an independent prognostic marker for STS. Compared with the patients in the high-risk group, those in the low-risk group exhibited a better prognosis and were more sensitive to several chemotherapeutic agents. In addition, the irlncRNA-based risk signature was significantly associated with immune scores, infiltrating immune cells, and the expression of several immune checkpoints.

**Conclusion:** In conclusion, our data revealed that the irlncRNA-based risk signature resulted in reliable prognosis, effectively predicted the immune landscape of patients with STS and was significantly correlated with chemosensitivity, thus providing insights into the potential role of irlncRNAs as prognostic biomarkers and novel therapeutic targets for STS.

## Introduction

Soft tissue sarcomas (STS), a rare and heterogeneous group of human malignancies with mesenchymal origin (Bourcier et al., 2019; Ratan and Patel, 2016), although only account for about 1% in all human malignancies, consist of more than 50 different distinct subtypes, but they only account for about 1% in all human malignancies (Jo and Fletcher, 2014; Poon and Quek, 2018). Despite surgery, radiotherapy and neoadjuvant chemotherapy, approximately 50% of patients with STS develop distant metastases, and the 5-year survival rate of patients with advanced STS is as low as approximately 28% (Italiano et al., 2011; Kim et al., 2019). Therefore, it is necessary to identify novel targets and develop more effective therapeutic strategies to improve the prognosis of patients with STS.

Recently, the tumor immune microenvironment has been proven to play a regulatory role in tumor progression. It comprises complex components, including multiple infiltrating immune cell types, immune checkpoints, immune-related molecules and signaling pathways, which are all critically involved in cancer development and immunotherapy (Zhang et al., 2019). It can not only inhibit tumor progression by killing tumor cells but also promote tumor progression by establishing conditions that facilitate the growth of tumor cells (Schreiber et al., 2011). For instance, cancer cells can evade immune system attack through immune checkpoints that inhibit immune functions, thus contributing to immune tolerance (Abril-Rodriguez and Ribas, 2017). Cancer immunotherapy has exhibited remarkable efficacy in cancer treatment over the past decades. Recently, multiple immune checkpoint inhibitors have been used for the treatment of several cancers, such as melanoma, renal cancer and lung cancer (Abril-Rodriguez and Ribas, 2017). Although immunotherapy has also been used for STS treatment in several clinical trials, its therapeutic efficacy and prolonged effects require further improvement (Ayodele and Razak, 2020). Therefore, further investigation should focus on exploring promising immune-related biomarkers and developing effective immunotherapeutic strategies for patients with STS. Moreover, numerous studies have investigated the promising potential of immunotherapy based on complex mechanisms that control the tumor immune microenvironment, such as diverse cellular components, signaling pathways and epigenetic modifications (Nguyen and Spranger, 2020; Rusek et al., 2015). However, the detailed regulatory networks of the tumor immune microenvironment have not been conclusively identified.

Long non-coding RNAs (lncRNAs) are a class of RNA transcripts longer than 200 nucleotides that do not code for proteins (Yang et al., 2019). Increasing evidence has revealed that lncRNAs play an important role in multiple physiological processes and human diseases, including human malignancies (Luo et al., 2021; Ransohoff et al., 2018; Schmitz et al., 2016). It has been found that a large number of lncRNAs are dysregulated during cancer progression, and multiple lncRNAs can serve as oncogenic or tumor suppressor genes in several cancers (Bhan et al., 2017; Liang et al., 2020). They can function as fundamental regulators of immune cell activities and immune-related gene expression, thereby modulating the tumor immune microenvironment in human malignancies (Huang et al., 2018). For instance, lncRNA SATB2-AS1, which was downregulated in colorectal cancer tissues, was correlated with tumor immune cell infiltration and affected tumor immune response by targeting SATB2 (Xu et al., 2019). In diffuse large B-cell lymphoma (DLBCL), SNHG14 can stimulate crosstalk between CD8+ T cells and DLBCL cells and induce CD8+ T cells by directly targeting the immune checkpoints PD-1/PD-L1 (Zhao et al., 2019). Recently, several studies have reported that immune-related lncRNAs (irlncRNAs) are correlated with the prognosis and clinicopathological parameters of patients with human malignancies and may facilitate the prognosis of multiple cancers (Wang et al., 2021b, a; Xu et al., 2021). However, the role of irlncRNAs in STS remains largely unknown, and no study has investigated the prognostic and clinicopathological value of irlncRNAs in patients with STS.

Therefore, our study was aimed to explore the association of irlncRNAs with prognosis, immune characteristics and chemosensitivity of STS for identifying the potential of irlncRNAs as prognostic biomarkers and novel therapeutic targets for STS.

## Materials and Methods

### Dataset

RNA-seq data were acquired from The Cancer Genome Atlas Sarcoma (TCGA-SARC) and Genotype-Tissue Expression (GTEx) datasets from the Genomic Data Commons (GDC) using the UCSC Xena browser^1^ (Goldman et al., 2015). The corresponding demographics (age and gender) and clinical characteristics (survival status, overall survival time, histological type, margin status, metastasis status, and confirmed recurrence and radiation therapy) were obtained from the TCGA-SARC database. The GTEx dataset provided RNA-seq data of 54 non-diseased tissue samples from approximately 1000 individuals. RNA-seq data were eventually collected from 78 normal soft tissue samples and 259 tumor samples from 259 patients. RNA-seq data from both TCGA-SARC and GTEx datasets were normalized to FPKM values for further investigation.

### Annotation of Long Non-coding RNAs

The lncRNA annotation file of Genome Reference Consortium Human Build 38 (GRCh38) was obtained from GENCODE^2^ to annotate lncRNAs of the gene expression files downloaded from the TCGA and GTEx datasets. A total of 14,081 lncRNAs identified in both datasets were enrolled in this study.

### Identification of Immune-Related Long Non-coding RNAs

A total of 2483 immune-related genes were extracted from the ImmPort database^3^ (Supplementary Material). Pearson correlation analysis was implemented to confirm irlncRNAs (with | Pearson R| > 0.5 and *P* < 0.001), and 1088 lncRNAs were considered as irlncRNAs. Subsequently, differentially expressed irlncRNAs (DEirlncRNAs) were extracted through differential analyses between 259 tumor and 78 normal samples using the “limma” R package. The expression differences were evaluated based on their log2 fold change (log2 FC) and false discovery rate (FDR), with the thresholds set at | log2FC| > 2 and FDR < 0.05. A total of 589 irlncRNAs were confirmed as DEirlncRNAs.

### Construction of Differently Expressed Immune-Related Long Non-coding RNAs Pairs

Differently Expressed irlncRNAs were paired using a loop-iteration method, and a 0-or-1 matrix was established by assigning C for each DEirlncRNA pair based on the expression level of both lncRNA A and lncRNA B: If the expression level of lncRNA A was higher than that of lncRNA B, C was bound to 1; otherwise, C was bound to 0. Subsequently, the established 0-or-1 matrix was checked ulteriorly. A DEirlncRNA pair was considered to have no prognostic value if the expression level of lncRNA pairs was 0 or 1 because pairs without a certain rank did not effectively predict the prognosis. If the value of a DEirlncRNA pair as 0 or 1 accounted for more than 80% samples enrolled in our study, it was considered to have no relationship with the prognosis of STS. Therefore, DEirlncRNA pairs with values 0 or 1 were selected for further investigation if they constituted more than 20% and less than 80% samples.

### Construction and Validation of Risk Signature

A total of 259 STS patients were randomly divided into a training cohort and a validation cohort at a ratio of 1:1 in R, including 130 patients in the training cohort and 129 patients in the validation cohort. The risk model construction was performed in the training cohort, and external validation in the validation cohort was critical when establishing the risk model. Univariate Cox regression analysis was conducted for profiling the prognostic DEirlncRNA pairs. The least absolute shrinkage and selection operator (LASSO) analysis was performed to identify the most significant prognostic DEirlncRNA pairs for constructing a risk signature employing the ‘glmnet’ R package. LASSO analysis was performed for 1000 iterations, and random stimulation was performed 1000 times in each iteration. The time required by each DEirlncRNA pair enrolled in the risk model for 1000-iteration LASSO analysis was recorded, and DEirlncRNA pairs that were recorded >100 times were included in the risk signature for further investigation. The risk score for each patient with STS was calculated based on the coefficients of every DEirlncRNA pair using the following formula: $R\,i\,s\,k\,S\,c\,o\,r\,e=\sum_{i=0}^{n}\beta_{i}*G\,i$. The area under the curve (AUC) values of each risk model were calculated to determine the optimal risk signature. When the maximum AUC value was reached, the calculation procedure was terminated. The predictive ability of the risk signature for 1-/3-/5-year overall survival was assessed using the “survivalROC” R package. In addition, the Akaike Information Criterion (AIC) value of each point on the receiver operating characteristic (ROC) curve for estimating the 5-year overall survival was calculated to find the maximum inflection point, which was identified as the cut-off value of the risk scores. All patients were then categorized into the high-risk and low-risk groups based on the cut-off value identified in the training set. Kaplan–Meier (K-M) survival curves along with the log-rank test were used to identify differences in overall survival between the two groups using the R packages “survival” and “survminer.” As for the validation of the risk signature, all the above analyses were conducted to assess the risk signature in the validation cohort.

To further investigate the clinicopathological significance of the risk signature, the chi-square test was performed to explore the relationship between the risk signature and clinicopathological characteristics using the R package “ComplexHeatmap.” The results were visualized on a band diagram, and statistical significance was labeled as follows: *P* < 0.001 = ^∗∗∗^, *P* < 0.01 = ^∗∗^, and *P* < 0.05 = ^∗^. We further used the Wilcoxon signed-rank test to assess differences in the risk scores among subgroups based on clinicopathological characteristics, and the results were visualized on a box diagram.

### Investigation of Immune Characteristics

The abundance of immune cells was analyzed using XCELL (Aran et al., 2017; Aran, 2020), TIMER (Li et al., 2017; Li T. et al., 2020), QUANTISEQ (Finotello et al., 2019; Plattner et al., 2020), MCPcounter (Dienstmann et al., 2019), EPIC (Racle et al., 2017), CIBERSORT-ABS (Tamminga et al., 2020), and CIBERSORT (Chen et al., 2018; Zhang et al., 2020). The immune, stromal and microenvironment scores were analyzed using XCELL. The relationship between the risk signature and infiltrating immune cells was analyzed using Spearman correlation analysis, and the correlation coefficients were visualized on a bubble diagram. The differences in immune cell infiltration between the high- and low-risk groups were analyzed using the Wilcoxon signed-rank test and were visualized using boxplots. The procedure was conducted using the R packages “ggplot2” and “ggpur.”

### Investigation of Chemotherapeutic Efficacy

The half maximal inhibitory concentration (IC50) of chemotherapeutic agents was calculated to assess the capability of the irlncRNA-based risk signature in predicting the chemotherapeutic efficacy in patients with STS. The Wilcoxon signed-rank test was used to compare IC50 between the high- and low-risk groups. The procedure was conducted and the results were visualized using the “pRRophetic” and “ggplot2” R packages.

### Statistical Analysis

R version 4.0.2 was used for statistical analyses and visualization. Limma package version 3.44.3 K-M survival curves along with the log-rank test were employed to evaluate the survival differences between the two groups. Univariate and multivariate Cox regression analyses were conducted to validate the independent role of the risk signature in the prognosis of patients with STS. Time-dependent ROC analysis was performed for assessing the predictive significance of the risk signature. A *P* value < 0.05 was considered statistically significant.

## Results

### Identification of Differently Expressed Immune-Related Long Non-coding RNAs

**FIGURE 1:**
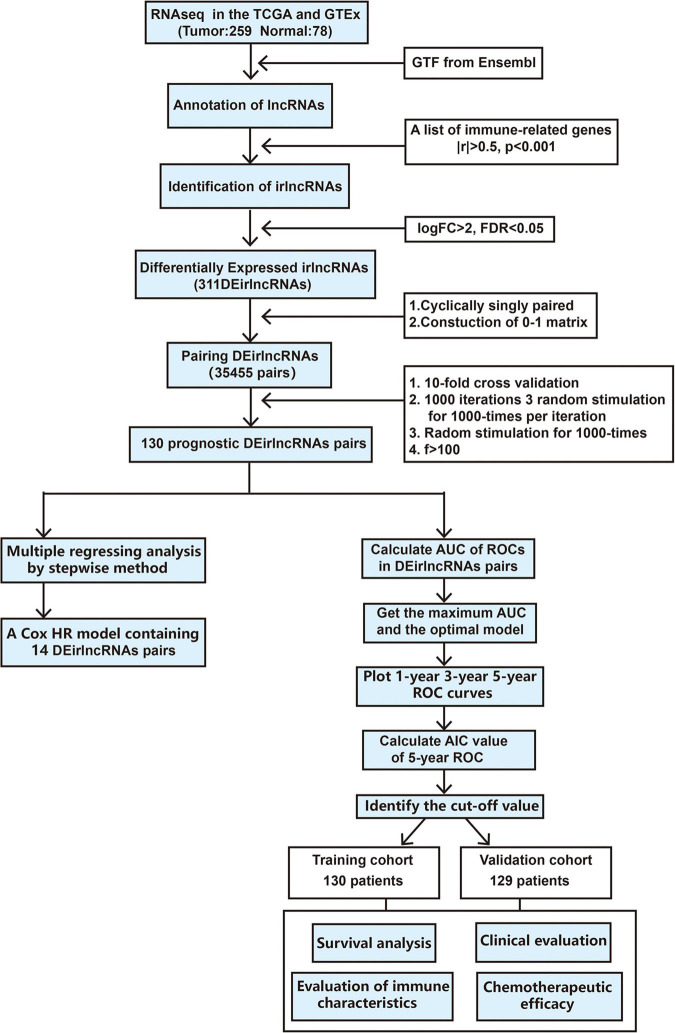
Flowchart of this study.

The procedure of our study is demonstrated in Figure 1. We extracted the gene expression profiling data, including 78 normal and 259 tumor samples, from the TCGA and GTEx datasets. The clinicopathological characteristics of patients with STS included in the analysis are provided in Table 1. A total of 259 patients with STS were enrolled in our study, including 58 with dedifferentiated liposarcoma (DDLPS), 104 with leiomyosarcoma (LMS), 25 with myxofbrosarcoma (MFS), 10 with synovial sarcoma (SS), 51 with undifferentiated pleomorphic sarcoma (UPS), and 11 with other STS types. Subsequently, we identified 14,081 lncRNAs in both datasets and extracted the expression matrices of 2483 immune-related genes from the datasets. We further conducted Pearson correlation analysis to identify irlncRNAs. The lncRNAs that were associated with one or more immune-related genes (| Pearson R| > 0.5 *and P* < 0.001) were defined as irlncRNAs. A total of 1088 lncRNAs were confirmed as irlncRNAs. Furthermore, 311 irlncRNAs were distinguished as DEirlncRNAs, including 45 upregulated and 266 downregulated irlncRNAs, by examining the expression profiles of 259 tumor tissues and 78 normal tissues (Supplementary Table 1). The mostly differentially expressed 100 DEirlncRNAs were visualized on the heatmap and volcano plot (Supplementary Figure 1).

**TABLE 1:** Clinical characteristics of STS patients in the training set and the validation set.

**Characteristics (*n*; %)**	**TGCA-SARC (*n* = 259)**	**Training set (*n* = 130)**	**Validation set (*n* = 129)**
**Age**			
≤60	128 (49.4)	67 (51.5)	61 (47.3)
>60	131 (50.6)	63 (48.5)	68 (52.7)
**Gender**			
Male	118 (45.6)	62 (47.7)	56 (43.4)
Female	141 (54.4)	68 (52.3)	73 (56.6)
**Histological type**			
DDLPS	58 (22.4)	27 (20.8)	31 (24.0)
LMS	104 (40.2)	50 (38.5)	54 (41.8)
MFS	25 (9.6)	12 (9.2)	13 (10.1)
SS	10 (3.9)	4 (3.1)	6 (4.7)
UPS	51 (19.7)	29 (22.3)	22 (17.1)
Other	11 (4.2)	8 (6.1)	3 (2.3)
**Metastasis**			
Yes	56 (21.6)	28 (21.5)	28 (21.7)
No	120 (46.3)	58 (44.6)	62 (48.1)
Unknow	83 (32.0)	44 (33.9)	39 (30.2)
**Margin status**			
Positive	73 (28.1)	37 (28.5)	36 (27.9)
Negative	136 (52.4)	68 (52.3)	68 (52.7)
Unknow	50 (19.3)	25 (19.2)	25 (19.4)
**Recurrence**			
Yes	29 (11.2)	14 (10.8)	15 (11.6)
No	143 (55.2)	72 (55.4)	71 (55.1)
Unknow	87 (33.6)	44 (33.8)	43 (33.3)
**Radiotherapy**			
Yes	74 (28.6)	45 (34.6)	29 (22.5)
No	179 (69.1)	83 (63.9)	96 (74.4)
Unknow	6 (2.3)	2 (1.5)	4 (3.1)

*A total of 259 patients with STS were enrolled in our study, including 58 with DDLPS, 104 with LMS, 25 with MFS, 10 with SS, 51 with UPS, and 11 with other STS types. They were randomly divided into the training set (130 patients), and the validation set (129 patients). DDLPS, dedifferentiated liposarcoma; LMS, leiomyosarcoma; MFS, myxofibrosarcoma; SS, synovial sarcoma; UPS, undifferentiated pleomorphic sarcoma.*

### Construction of Differently Expressed Immune-Related Long Non-coding RNAs Pairs and a Risk Signature

**FIGURE 2:**
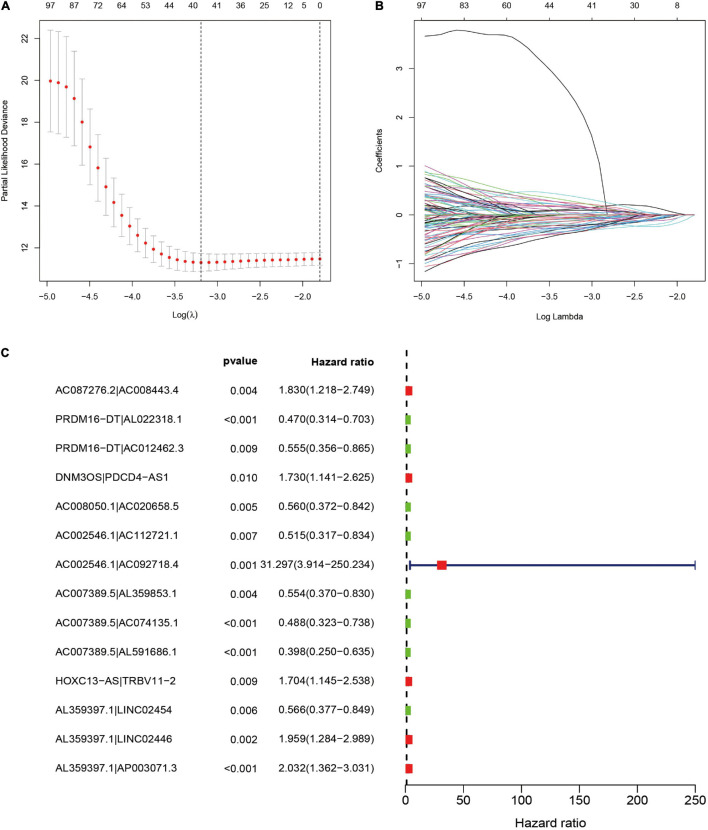
Selection of DEirlncRNA pairs for the risk signature. **(A,B)** LASSO analysis with minimal lambda value. **(C)** Forest plot of the prognostic ability of 14 DEirlncRNA pairs identified by Cox proportional hazard regression included in the risk signature by the stepwise method.

**FIGURE 3:**
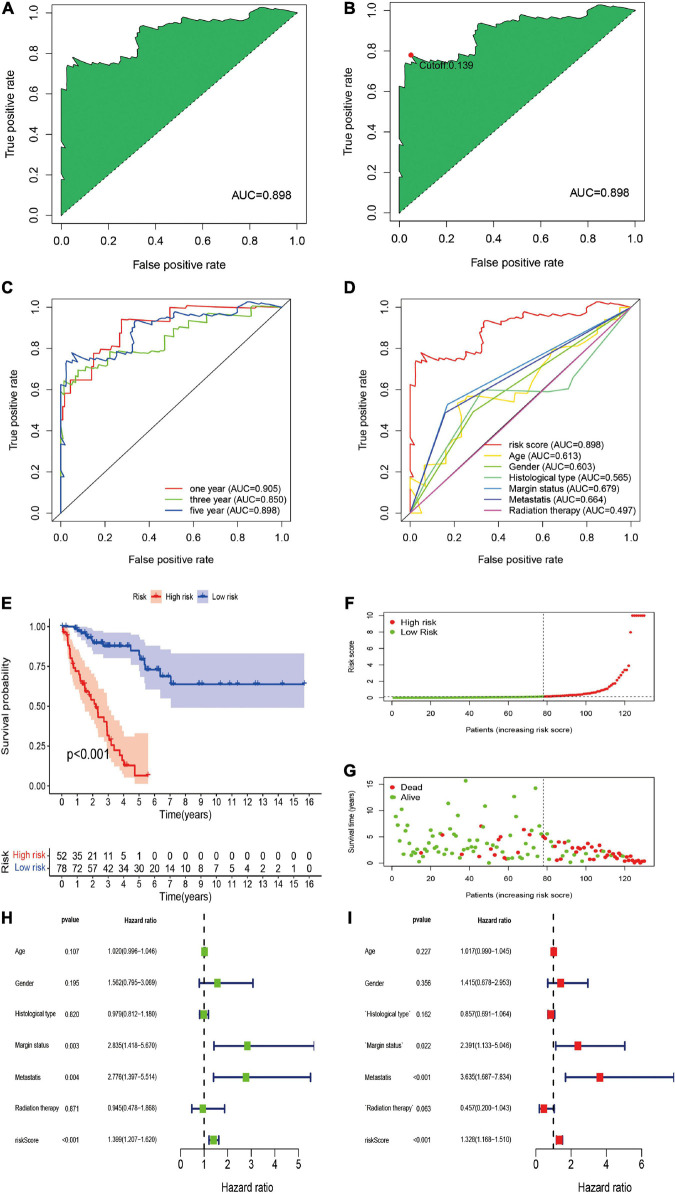
Construction of DEirlncRNA pairs-based risk signature in the training cohort. **(A)** The ROC of the optimal DEirlncRNA pairs-based risk signature with the highest AUC. **(B)** Identification of the cut-off point on the ROC curve by the AIC. **(C)** The 1-, 3-, and 5-year ROC curves of the optimal model. **(D)** Comparison of the 5-year ROC curve with other clinical characteristics. **(E)** Kaplan–Meier analysis of patients in the high risk and low risk groups. Patients in the low-risk group experienced a longer survival time. **(F,G)** Distributions of risk scores and survival status of STS patients. **(H,I)** Univariate and multivariate Cox regression analyses of clinical factors and prognostic risk signature for overall survival. Univariate and multivariate analyses revealed that risk score was an independent prognostic predictor.

To further establish DEirlncRNA pairs among 311 DEirlncRNAs, we used a loop-iteration method and constructed a 0-or-1 matrix. As a result, a total of 35455 valid DEirlncRNA pairs were established. Subsequently, we selected 130 prognostic DEirlncRNA pairs using univariate Cox regression analysis. By conducting modified LASSO Cox analysis based on 130 prognostic DEirlncRNA pairs, 40 DEirlncRNA pairs were employed to establish a risk assessment model, and 14 pairs were further enrolled in a Cox proportional hazard model using the stepwise method (Figures 2A–C). All patients were divided into the following two cohorts: 130 in the training cohort and 129 in the validation cohort. Furthermore, we calculated the 5-year AUC values of each model to identify the maximum value for an optimal model, and the result indicated that the highest 5-year AUC value was 0.898 (Figure 3A). Using these AIC values, the maximum inflection point on the 5-year ROC was identified as the cut-off value (Figure 3B). All patients with STS in the training cohort were divided into the following two groups based on the cut-off value: 54 patients in the high-risk group and 76 in the low-risk group. Subsequently, we calculated AUC values to assess the performance of the nomogram in predicting the overall survival of patients in the training cohort. The AUC values of 1-, 3-, and 5-year survival were 0.905, 0.850, and 0.898, respectively (Figure 3C). Furthermore, we compared the AUC values of 5-year survival of the risk signature with other clinicopathological features, and the results revealed that the risk signature was more accurate than other predictors (Figure 3D). K-M analysis was employed for assessing the differences in overall survival between the high- and low-risk groups. The result revealed that high-risk patients had a remarkably shorter survival time than that of low-risk patients *(P <* 0.001; Figure 3E). The risk scores and survival status of each patient are shown in Figures 3F,G; patients in the high-risk group had worse clinical outcomes. In addition, the results of univariate Cox analysis suggested that the risk signature was significantly correlated with overall survival (hazard ratio [HR], 1.399; 95% confidence interval [CI], 1.207–1.620; *P* < 0.001; Figure 3H). The results of multivariate Cox analysis demonstrated that the risk signature functioned as an independent prognostic marker for patients with STS (HR, 1.328; 95% CI, 1.168–1.510; *P* < 0.001; Figure 3I). Univariate Cox analysis also revealed that the margin status (HR, 2.835; 95% CI, 1.418–5.670; *P* = 0.003; Figure 3H), and the metastasis status (HR, 2.776; 95% CI, 1.397–5.514; *P* = 0.004; Figure 3H) was closely associated with the prognosis of patients, and multivariate Cox analysis further indicated that the margin status (HR, 2.391; 95% CI, 1.133–5.046; *P* = 0.022; Figure 3I), and the metastasis status (HR, 3.635; 95% CI, 1.687–7.834; *P* < 0.001; Figure 3I) functioned as an independent prognostic variable.

### Validation of Immune-Related Long Non-coding RNAs-Based Risk Signature

**FIGURE 4:**
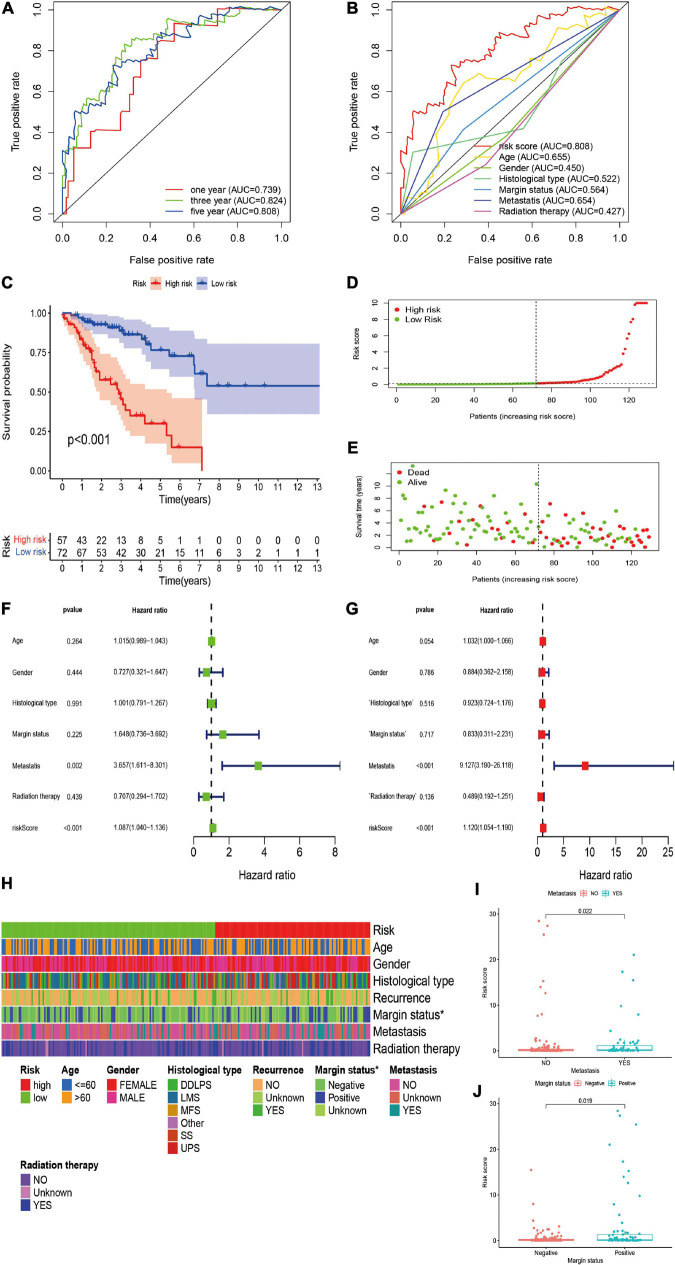
Validation and clinical evaluation of the irlncRNA-based risk signature. **(A)** The 1-, 3-, and 5-year ROC curves of the risk signature in the validation cohort. **(B)** Comparison of the 5-year ROC curve with other clinical characteristics. **(C)** Kaplan–Meier analysis of patients in the high risk and low risk groups. Patients in the low-risk group experienced a longer survival time. **(D,E)** Distributions of risk scores and survival status of STS patients. **(F,G)** Univariate and multivariate Cox regression analyses of clinical factors and prognostic risk signature for overall survival. Univariate and multivariate analyses revealed that risk score was an independent prognostic predictor in the validation cohort. **(H–J)** Evaluation of the association between the risk signature and clinical parameters. A strip chart **(H)** along with the scatter diagram showed that **(I)** metastasis, and **(J)** margin status were significantly correlated with the risk score.

We further validated the predictive value of the risk signature in the independent validation cohort. A total of 129 patients with STS were included in the independent validation cohort. The 1-/3-/5-year AUC values were 0.739, 0.824, and 0.808, respectively, which indicated the satisfactory performance of the risk signature in the cohort (Figure 4A). In addition, the 5-year AUC value of the risk signature was also significantly higher than that of other clinicopathological variables, indicating that the risk signature was more accurate than other predictors (Figure 4B). Survival analysis indicated that the overall survival of high-risk patients was significantly worse than that of their low-risk counterparts (*P* < 0.001; Figure 4C). The risk score and survival status plots of patients demonstrated that the survival time and survival rate were decreased with an increasing risk score (Figures 4D,E). Moreover, univariate analysis confirmed the significant correlation between risk scores and prognosis (HR, 1.087; 95% CI, 1.040–1.015; *P* < 0.001; Figure 4F), and multivariate analysis further confirmed the role of the risk score as an independent prognostic factor in STS (HR, 1.120; 95% CI, 1.054–1.190; *P* < 0.001; Figure 4G). In conclusion, all these results were consistent with those of the training cohort, thus verifying the prognostic significance of the risk model in STS.

### Analysis of Clinical Parameters Using the Risk Signature

To further verify the clinicopathological significance of the irlncRNA-based risk signature, we conducted chi-square tests to evaluate the correlation between the risk scores and clinicopathological characteristics of patients with STS. The heatmap demonstrated that the risk score might be correlated with the margin status of patients, and the scatter diagrams obtained by the Wilcoxon signed-rank test identified that the high-risk score was significantly associated with metastasis (*P* = 0.0022), and positive margin status (*P* = 0.0019) (Figures 4H–J).

### Correlation of the Risk Signature With Immune Characteristics

**FIGURE 5:**
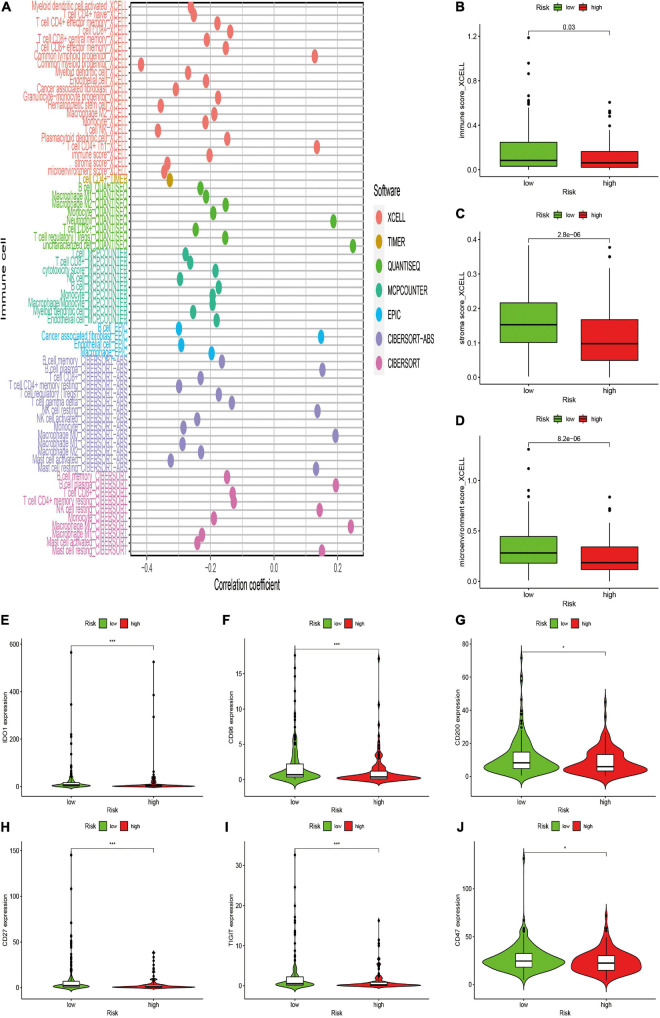
Estimation of the associations between immune characteristics and the risk signature. **(A)** Diagram of Spearman correlation analysis between immune cells infiltration and the risk signature. Patients in the high-risk group were more positively associated with tumor-infiltrating immune cells such as macrophage M0, and NK resting cells, whereas they were negatively associated with CD8+ T cells, CD4+ T cells, and monocytes. **(B–D)** The correlation between the risk signature and immune score by XCELL. Patients in the high-risk group had a lower **(B)** immune, **(C)** stromal scores, and **(D)** microenvironment scores. **(E–J)** The expression levels of immune checkpoints in the high-risk and low-risk groups. High risk scores were negatively correlated with upregulated **(E)** IDO1, **(F)** CD96, **(G)** CD200, **(H)** CD27, **(I)** TIGIT, and **(J)** CD47 levels.

After constructing the irlncRNA-based risk signature, we further assessed its association with immune characteristics in STS. The results of the Wilcoxon signed-rank test revealed that the high-risk score was positively correlated with infiltrating immune cells such as macrophage M0, resting mast cells and resting natural killer (NK) cells, whereas the high-risk score was negatively correlated with CD4+ T cells, CD8+ T cells, monocytes, macrophage M1 and memory B cells (Supplementary Figure 2). Currently acknowledged methods including XCELL, TIMER, QUANTISEQ, MCPcounter, EPIC, CIBERSORT-ABS and CIBERSORT were employed to analyze the relationship between the abundance of infiltrating immune cells and risk scores, and the results of Spearman correlation analysis were demonstrated on a lollipop-shaped diagram (Figure 5A and Supplementary Table 2). In addition, the low-risk group had higher immune, microenvironment and stromal scores, which were evaluated using XCELL (Figures 5B–D). In addition, the risk score exhibited a significant correlation with the expression of several immune checkpoints. The high-risk group exhibited lower expression of IDO1, CD96, CD200, CD27, TIGIT, and CD47 than that exhibited by the low-risk group (Figures 5E–J). These results indicated that the risk signature was closely related to the abundance of immune cells and microenvironment scores and might predict immunotherapeutic efficacy in patients with STS.

### Correlation of the Risk Signature With Chemotherapeutic Efficacy

**FIGURE 6:**
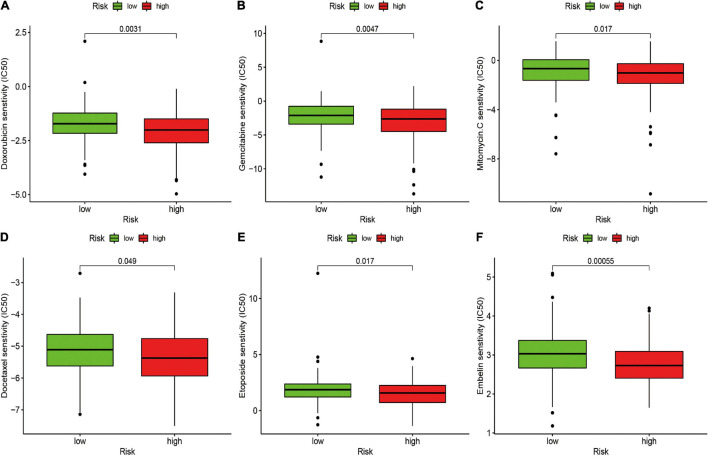
Estimation of the Associations between Chemosensitivity and the Risk Signature. The ICI50 of **(A)** doxorubicin, **(B)** gemcitabine, **(C)** mitomycin C, **(D)** docetaxel, **(E)** etoposide, and **(F)** embelin in the high-risk and low-risk groups. The risk signature acted as a potential predictor for chemosensitivity as high-risk scores were associated with a lower IC50 for chemotherapeutics including doxorubicin, gemcitabine, mitomycin C, docetaxel, etoposide, and embelin.

The potential of the risk signature in predicting chemotherapeutic efficacy in patients with STS was also investigated. The results revealed that a low-risk score was significantly related to higher IC50 of several chemotherapeutics, including doxorubicin (*P* = 0.0031), gemcitabine (*P* = 0.0047), mitomycin C (*P* = 0.017), docetaxel (*P* = 0.049), etoposide (*P* = 0.017), and embelin (*P* = 0.00055), indicating that the irlncRNA-based risk signature effectively predicted chemotherapeutic efficacy in patients with STS (Figure 6).

## Discussion

The present study is the first of its kind to construct a novel irlncRNA-based risk signature and comprehensively investigate the potential role of irlncRNAs in STS. In this study, 259 patients were enrolled from the TCGA dataset; 35455 DEirlncRNA pairs were constructed, and 130 pairs were further confirmed as prognostic markers for STS. Of the 130 pairs, 14 were selected to construct the irlncRNA-based risk signature. Several lncRNAs, such as LINC02454, lncRNA PDCD4-AS1 and LINC02446, used in the risk signature have been reported to play a critical role in cancer progression. For instance, LINC02454 was previously reported to be upregulated in papillary thyroid cancer, was closely related to various clinicopathological features, including large tumor size, advanced clinical stage and lymph node metastasis and served as a diagnostic and prognostic biomarker for papillary thyroid cancer (Cai et al., 2018; Li Z. et al., 2021; Tan et al., 2020). Via sponging miR-10b-5p to upregulate *IQGAP2*, lncRNA PDCD4-AS1, a downregulated in both triple-negative breast cancer tissues and cell lines and can suppress tumor progression (Jadaliha et al., 2018; Wang et al., 2021c). In addition, via inhibiting the mTOR signaling pathway by directly binding to the EIF3G protein, exogenous overexpression of LINC02446 could inhibit cellular proliferation, migration and invasion in bladder cancer (Zhang et al., 2021). It has been found that LncRNA HOXC13-AS can serve as an oncogene in multiple human malignancies (Li X. et al., 2019; Li W. et al., 2020; Liu et al., 2019). For example, HOXC13-AS has been reported to promote the proliferation of breast cancer cells and tumor growth via epigenetically inhibiting PTEN expression by sponging miR-497-5p in both vivo and vitro (Li X. et al., 2019). However, further investigation is required because only a few studies have investigated the role of these irlncRNAs in STS.

Previous studies have focussed on the potential role of irlncRNAs in cancer progression and constructed risk models based on irlncRNAs with promising predictive significance in various cancers (Wang et al., 2021b). To the best of our knowledge, these risk models were mostly based on the exact expression levels of irlncRNAs. In this study, we established a novel irlncRNA-based algorithm for patients with STS that did not require the exact expression of irlncRNAs. This novel algorithm only required the determination of irlncRNA pairs with either higher or lower expression levels instead of the exact expression levels of irlncRNAs, which minimized sample errors caused by varying expression levels and made our risk model more reliable and convenient. We first confirmed DEirlncRNAs using a differential co-expression analysis and paired DEirlncRNAs using an improved loop-iteration method with a 0-or-1 screening matrix. Furthermore, we selected the most remarkable DEirlncRNA pairs for constructing the novel algorithm using a modified Lasso penalized modeling method to improve the prognostic potential and facilitate the clinical application of the risk signature (Sveen et al., 2012). This modified Lasso penalized modeling method mainly included cross-validation, multiple repeats and random stimulation. It was conducted by incorporating the factors into a Cox regression model based on the rank of occurrence frequency instead of the intersection of occurrence because frequency can reveal the effectiveness of the factor. Moreover, we used an improved method to construct the risk model as follows: Every AUC value of each risk model was calculated to identify the maximum value and confirm the most ideal DEirlncRNA pair for establishing the risk signature. In addition, instead of the median value, the AIC value was identified as the cut-off point for distinguishing the subgroups because it is superior to conventional prognostic models in dividing patients into the high- and low-risk groups. The time-dependent AUC values of 5-year overall survival were higher than 0.80 and the AUC value of the risk signature was significantly higher than that of other clinical characteristics, indicating the reliable predictive ability and superiority of our irlncRNA-based risk model. Both univariate and multivariate regression analyses were further conducted, which revealed that the risk signature functioned as an independent prognostic marker for patients with STS. In addition, the high-risk group exhibited worse overall survival, and the risk signature was associated with metastasis and histological types of STS. These findings confirmed the prognostic and clinicopathological value of the irlncRNA-based risk signature in STS.

As expected, we found that the irlncRNA-based risk signature was significantly associated with the tumor immune microenvironment of STS. The immune, stromal and microenvironment scores calculated using XCELL were significantly higher in the low-risk group. Previous studies have found that stromal and microenvironment scores calculated using XCELL were positively associated with a reliable prognosis of patients with cancer (Deng et al., 2019; Zou et al., 2020). To examine the association between risk scores and infiltrating immune cell types, seven common acceptable methods were employed for estimating the abundance of infiltrating immune cells, including XCELL, TIMER, QUANTISEQ, MCPcounter, EPIC, CIBERSORT-ABS, and CIBERSORT. Owing to the complexity and defects of these methods, we further performed an integrating analysis for each infiltrating immune cell type. The results demonstrated that the low-risk group exhibited a higher infiltration level of immune cells including CD4 + T cells, CD8 + T cells, activated mast cells, macrophage M1, monocytes and activated NK cells and a lower infiltration level of activated memory CD4 + T cells, resting NK cells and macrophage M0. Infiltrating immune cells in the tumor immune microenvironment are critically involved in cancer initiation and development and exhibit a significant correlation with the clinical outcome of patients. Previous studies have revealed that a higher abundance of macrophage M0 was significantly correlated with worse prognosis and clinical outcomes, whereas an abundance of macrophage M1 led to a reliable prognosis in several cancers. Our results were consistent with those of previous studies (Huang et al., 2019; Yuan et al., 2015). Furthermore, it has been reported that the infiltration of CD4 + and CD8 + T cells can be regarded as a reliable prognostic factor in sarcomas (Ostroumov et al., 2018). Moreover, an abundance of CD4 + and CD8 + T cells plays an important role in tumor response to immunotherapies. Alspach et al. have reported that the anti-tumor efficacy of immune checkpoint blockade therapy requires the activation of CD4 + and CD8 + T cells in sarcomas (Alspach et al., 2019). In conclusion, our risk signature was significantly associated with immune cell infiltration and confirmed its correlation with the prognosis of patients with STS.

Targeting immune checkpoints provide novel insights into cancer therapy, and several immune checkpoint inhibitors, such as PD-1/PD-L1 and CTLA-4 inhibitors, have been used for the treatment of multiple cancers, including melanoma, kidney cancer and non-small cell lung cancer (Li B. et al., 2019; Postow et al., 2018). Recently, the efficacy of immune checkpoint inhibitors in STS was investigated in multiple clinical trials; however, these trials reported controversial results. The low response rate to immune checkpoint inhibitors in some patients with STS may be a major hurdle in improving immunotherapeutic efficacy (Zhu et al., 2020). Therefore, effective biomarkers that can predict response to immune checkpoint inhibitors in patients with STS should be developed, which may improve prognosis by enhancing the efficacy of the inhibitors. In this study, we investigated the potential role of irlncRNAs as predictors for immune checkpoint expression. The results revealed that the risk score was negatively correlated with the expression of IDO1, CD96, CD200, CD27, TIGIT, and CD47. Indoleamine 2,3-dioxygenase 1 (IDO1), an important immune checkpoint, is a rate-limiting metabolic enzyme that contributes to the conversion of the essential amino acid tryptophan (Trp) into kynurenines (Munn and Mellor, 2016). Emerging evidence indicates that IDO1 is highly expressed in various human malignancies and is important for regulating cancer progression and tumor immune microenvironment (Bishnupuri et al., 2019; Xiang et al., 2019). In addition, IDO1 exhibits great prognostic and clinicopathological significance for patients with cancer. However, the prognostic value is inconsistent among different cancers (Zhai et al., 2018). A study reported that high IDO1 expression was correlated with prolonged survival time in patients with UPS, which was consistent with our results (Ishihara et al., 2021). B7-H3 (CD276), a member of the B7-CD28 family, is also a crucial immune checkpoint member that can suppress the functions of T cells (Flem-Karlsen et al., 2020). It has been found that B7-H3 is upregulated in multiple human cancers, and B7-H3 overexpression is closely related to poor prognosis and clinical outcomes (Flem-Karlsen et al., 2020). Because of the low expression of B7-H3 in normal tissues, it has become a promising target for cancer immunotherapy, and several therapeutic strategies that target B7-H3, such as small-molecule inhibitors and chimeric antigen receptor T (CAR-T) cell technology, have been used for the treatment of cancer in clinical trials (Picarda et al., 2016). Recent studies have investigated CAR-T cell immunotherapy that targets B7-H3 in bone sarcoma models in vivo, with a significantly prolonged survival time. Therefore, B7-H3 CAR-T cell therapy may also be an effective immunotherapeutic strategy for patients with STS. Our study provided a promising biomarker for predicting the expression of immune checkpoints and immunotherapeutic efficacy in patients with STS. However, the exact expression pattern and potential role of these immune checkpoints in STS remain undefined. Comprehensive and in-depth studies are necessary for investigating the expression and role of these immune checkpoints and the capability of the irlncRNA-based risk signature to predict immunotherapeutic efficacy in patents with STS.

Although there has been advancement in chemotherapy for patients with STS in the past decades, many patients eventually develop intrinsic or acquired resistance to chemotherapeutic agents, thereby contributing to limited chemotherapeutic efficacy and a poor prognosis. Therefore, we further assessed the predictive value of the irlncRNA-based risk signature to assess chemosensitivity in patients with STS. The results revealed that the irlncRNA-based risk signature effectively predicted the response of patients to doxorubicin, gemcitabine, docetaxel, mitomycin C and embelin. Doxorubicin, an anthracycline antibiotic that can inhibit the synthesis of both DNA and RNA by embedding into DNA base pairs, has become the most effective and commonly used chemotherapeutic agent for patients with STS (Ratan and Patel, 2016). Owing to STS progression or the development of resistance to first-line chemotherapy, a combination of gemcitabine and docetaxel is used as a standard second-line treatment for patients with STS (Ratan and Patel, 2016). Therefore, our risk signature may function as a promising predictor of chemotherapeutic efficacy, thereby expanding new avenues for selecting the most suitable chemotherapy for each patient with STS.

However, this quality study also has several limitations. The findings are based on clinical samples downloaded from the GDC TCGA-SARC dataset instead of our cohort. The results of the present study should be further validated in patients of our cohort. Furthermore, the correlation between irlncRNAs and STS immune characteristics were not investigated in experiments, and the detailed mechanism by which irlncRNAs modulate STS immune microenvironment remains unknown. Lastly, the novel risk signature should be further validated in larger clinical samples before incorporating it into diagnostic and therapeutic practice.

## Conclusion

We comprehensively investigated the potential functions and clinical value of irlncRNAs in STS. We established a novel risk signature based on DEirlncRNA pairs for patients with STS. The novel risk signature was significantly correlated with immune characteristics and effectively predicted chemotherapeutic efficacy in patients with STS, thereby serving as a reliable prognostic factor. We identified the prognostic and clinicopathological value of irlncRNAs as well as the correlation between irlncRNAs and immune microenvironment in STS, thus highlighting the promising role of irlncRNAs as clinical biomarkers and novel therapeutic targets for patients with STS.

## Data Availability Statement

The datasets presented in this study can be found in online repositories. The names of the repository/repositories and accession number(s) can be found in the article/Supplementary Material.

## Author Contributions

ZL: writing – original draft, data curation, and formal analysis. KP and XZ: writing – original draft. HL and TZ: writing – review and editing, and visualization. JW: formal analysis. TL and WP: writing – review and editing, supervision, and funding acquisition. All authors contributed to the writing and revision of the manuscript, knew the content of it, and approved its submission.

## Conflict of Interest

The authors declare that the research was conducted in the absence of any commercial or financial relationships that could be construed as a potential conflict of interest.

## Publisher’s Note

All claims expressed in this article are solely those of the authors and do not necessarily represent those of their affiliated organizations, or those of the publisher, the editors and the reviewers. Any product that may be evaluated in this article, or claim that may be made by its manufacturer, is not guaranteed or endorsed by the publisher.

## Footnotes

1
https://xenabrowser.net/

2
https://www.gencodegenes.org/human/

3
https://immport.niaid.nih.gov

## Abbreviations

irlncRNAs: immune-related long non-coding RNAs
STS: soft tissue sarcomas
TCGA: The Cancer Genome Atlas
GTEx: genotype-tissue expression
DEirlncRNA: differently expressed irlncRNA
AUC: area under the ROC curve
lncRNAs: long non-coding RNAs
DLBCL: diffuse large B cell lymphoma
AIC: akaike information criterion
IC50: half inhibitory centration
GRCh38: Genome Reference Consortium Human Build 38
DDLPS: dedifferentiated liposarcoma
LMS: leiomyosarcoma
MFS: myxofibrosarcoma
SS: synovial sarcoma
UPS: undifferentiated pleomorphic sarcoma
SS: synovial sarcoma
IDO1, Indoleamine 2: 3-dioxygenase 1
Trp: tryptophan
CAR-T: chimeric antigen receptor T.
