# Supplementary material for: Characterization of Immune-Related Long Non-coding RNAs to Construct a Novel Signature and Predict the Prognosis and Immune Landscape of Soft Tissue Sarcoma
Source: Front Cell Dev Biol. 2021 Sep 24;9:709241. doi: 10.3389/fcell.2021.709241 (PMC8497898; doi:10.3389/fcell.2021.709241)
Supplement: Supplementary file 5 [file Table_5.DOCX]

**Supplementary Table and Figure Legends**

**Supplementary Tables:**

**Table S1:** List of samples from TCGA-SARC and GTEx

**Table S2:** List of 2483 immune-related genes

**Table S3:** List of 311 differentially expressed immune-related lncRNAs

**Table S4:** The detail comparison results of correlation ship between tumour infiltrating immune cells and risk sore

**Supplementary Figures:**

**Figure S1:** Identification of differentially expressed immune-related lncRNAs (DEirlncRNAs) between STS and normal samples. (A) Heatmap of DEirlncRNAs in STS and normal samples. (B) Volcano plot for DEirlncRNAs.

**Figure S2:** The representative results of the evaluation of tumor infiltrating immune cells with risk signature.
