# Supplementary figures and images for: Characterization of Immune-Related Long Non-coding RNAs to Construct a Novel Signature and Predict the Prognosis and Immune Landscape of Soft Tissue Sarcoma
Source: Front Cell Dev Biol. 2021 Sep 24;9:709241. doi: 10.3389/fcell.2021.709241 (PMC8497898; doi:10.3389/fcell.2021.709241)

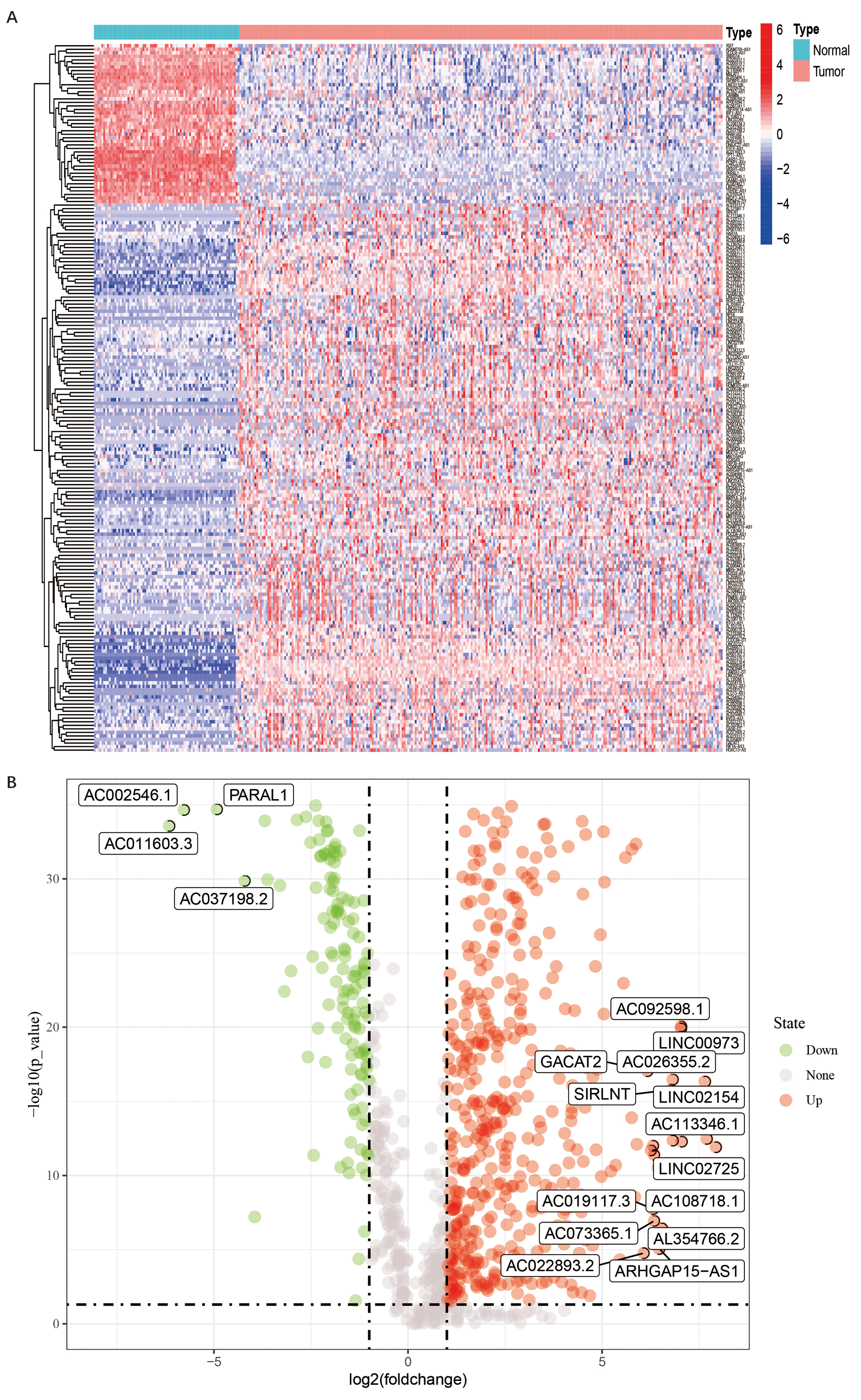

Supplement: Supplementary file 6 [file Image_1.JPEG]

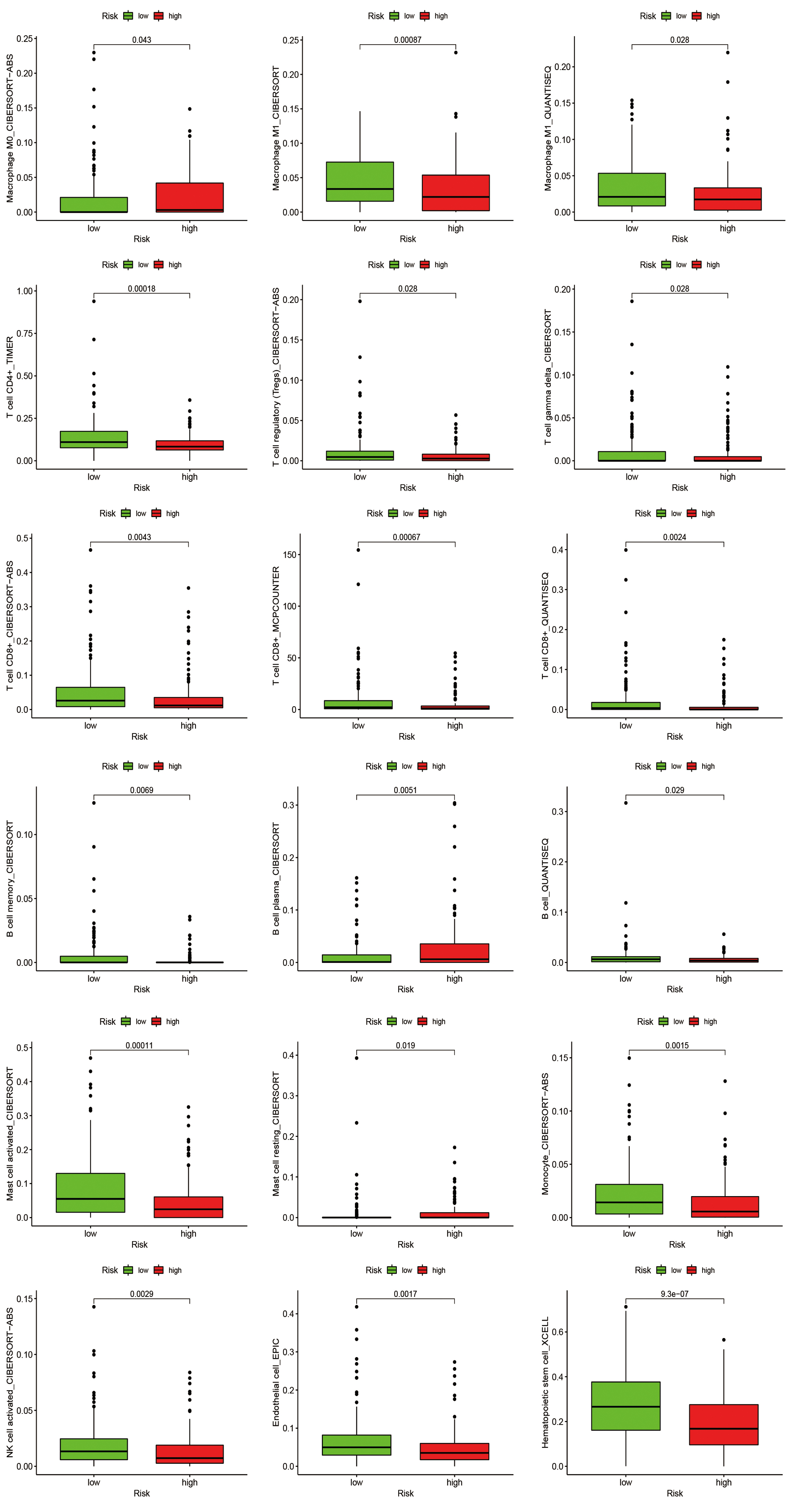

Supplement: Supplementary file 7 [file Image_2.JPEG]
